# The Ckd. Qld fabRy Epidemiology (aCQuiRE) study protocol: identifying the prevalence of Fabry disease amongst patients with kidney disease in Queensland, Australia

**DOI:** 10.1186/s12882-020-01717-9

**Published:** 2020-02-22

**Authors:** Andrew Mallett, Phoebe Kearey, Anne Cameron, Helen Healy, Charles Denaro, Mark Thomas, Vincent W. Lee, Samantha Stark, Maria Fuller, Wendy E. Hoy

**Affiliations:** 1 Kidney Health Service and Conjoint Renal Research Laboratory, Royal Brisbane and Women’s Hospital, Brisbane, Australia; 2 Institute for Molecular Bioscience, The University of Queensland, Brisbane, Australia; 3 Faculty of Medicine, The University of Queensland, Brisbane, Australia; 4 The KidGen Collaborative, Australian Genomic Health Alliance, Parkville, Australia; 5 CKD.QLD and NHMRC CKD.CRE, The University of Queensland, Brisbane, Australia; 6 Department of Renal Medicine, Royal Brisbane and Women’s Hospital, Level 9 Ned Hanlon Building, Butterfield Street, Herston, Queensland 4029 Australia; 7 Department of Internal Medicine and Aged Care, Royal Brisbane and Women’s Hospital, Brisbane, Australia; 8 Department of Nephrology, Royal Perth Hospital, Perth, Australia; 9 Department of Renal Medicine, Westmead Hospital, Sydney, Australia; 10 Sydney Medical School, Faculty of Medicine and Health, University of Sydney, Sydney, Australia; 11 Genetics and Molecular Pathology Laboratory (SA Pathology), Adelaide, Australia

**Keywords:** Fabry disease, Lysosomal storage disorder, Alpha-galactosidase, Genetic testing

## Abstract

**Background:**

Fabry disease (FD) is a rare, lysosomal storage disorder caused by the absence or deficiency of the enzyme alpha-galactosidase A (α-Gal A) that leads to the abnormal accumulation of the lipid globotriaosylceramide (GB3) in a variety of cell types and tissues throughout the body. FD has an x-linked inheritance pattern. Previously thought to be only carriers, females can also experience FD symptomatology. Symptoms vary in type and severity from patient to patient and tend to increase in severity with age. FD symptoms are non-specific and may be shared with those of other diseases. Misdiagnoses and diagnostic delays are common, often resulting in progressive, irreversible tissue damage. The estimated prevalence of FD in the general population is 1:40,000 to 1:117,000 individuals. However, it is estimated that the prevalence of FD in the dialysis population is 0.12 to 0.7%. Little is known about the prevalence of FD in the broader Chronic Kidney Disease (CKD) population.

**Methods:**

This is an epidemiological study of the prevalence of FD in CKD patents identified from the public renal speciality practices in Queensland, Australia. A cascade approach to screening is being employed with dried blood spot testing for blood levels of alpha-galactosidase A (Alpha-Gal), with follow-up testing for patients with abnormal results by plasma levels of globotriaosylsphingosine (Lyso-GB3) for females and non-definitive cases in males. A diagnosis of FD is confirmed through genetic testing of the GLA gene in cases suspected of having FD based upon Alpha-Gal and Lyso-GB3 testing.

**Discussion:**

Expected outcomes of this study include more information about the prevalence of FD at all stages of CKD, including for both males and females. The study may also provide information about common characteristics of FD to assist with diagnosis and optimal management/treatment. Screening is also available for family members of diagnosed patients, with potential for early diagnosis of FD and intervention for those individuals.

**Trial registration:**

Queensland Health Database of Research Activity (DORA, https://dora.health.qld.gov.au) pj09946 (Registered 3rd July 2017).

## Background

Fabry disease (FD) is a rare genetic condition and lysosomal storage disorder. It is caused by the absence or deficiency of the enzyme alpha-galactosidase A (α-Gal A) that leads to the accumulation of a number of glycosphingolipids in a variety of cell types and tissues throughout the body, with globotriaosylceramide (GB3) being the predominant species. Consequently, FD is a multisystem disorder affecting kidney, skin, cardiovascular and neurological systems.

Symptoms vary in type and severity from patient to patient though they tend to increase in severity with age [1]. Typical symptoms include acroparathesias, hypohydrosis or absence of sweating with associated heat intolerance, angiokeratomas, and vasculopathy of retina and/or conjunctiva [1]. More severe effects include renal [2–4], cardiac [2–4], and cerebrovascular systems manifestations [2, 4], which are major causes for morbidity and mortality amongst patients affected by FD [4, 5]. Other symptoms found to be associated with FD include: nausea, vomiting and diarrhoea; dyspnoea; auditory and vestibular symptoms such as hearing loss, vertigo, and tinnitus; lymphoedema; and osteopenia and osteoporosis [1]. Fabry disease has been found to be linked with poorer quality of life compared to the general population [6, 7], particularly for those with kidney manifestations, more severe disease and amongst older patients [6].

FD has an X-linked inheritance pattern with α-Gal A encoded by the *GLA* gene containing seven exons across 12 kb and located chromosomally at Xq22.1 [8, 9]. Whilst females were previously thought to be non-manifesting carriers, it has been shown that females also develop Fabry disease signs and symptomatology [10–12] albeit with greater variability of severity and age at onset compared to affected males [13]. In fact, 69.4 to 80% of females harboring a disease-causing *GLA* variant report some degree of symptoms [14, 15].

Whilst the greater majority of males affected by FD present with a classical phenotype, there are clinical FD variants that are defined in patients with predominant cardiac or renal involvement, who have the absence of other typical FD clinical manifestations [1, 16, 17]. In the renal variant of FD only kidney manifestations tend to present and do so later in life, typically in the 4th to 6th decades [1]. Patients with these atypical variants have residual α-gal A activity levels [18], most frequently due to missense or splicing *GLA* genetic variants [19]. The presence of residual endogenous α-Gal A enzyme is thought to be responsible for the lack of earlier clinical manifestations. Branton et al. [14] found that the earliest onset of renal symptoms in Fabry patients with residual alpha-Gal A activity was 47 years, compared to 22 years in those with no residual alpha-Gal A activity.

The estimated prevalence of FD in the general population is only 1:40,000 (0.0025%) [20] to 1:117,000 (0.0009%) individuals [21]. A systematic review of Fabry screening identified six newborn studies with an overall estimated population prevalence of 0.04% (ranging from 0.0 to 0.04%) [22]. Two subsequent large-scale newborn screening programs for lysosomal storage disorders (LSDs) conducted in the USA estimated the population prevalence at 0.01% [23, 24] and a Japanese Fabry screening pilot also estimated population prevalence to be 0.01% [25]. Three additional large-scale studies examined the prevalence of Fabry disease in male neonates [12, 26, 27], with Fabry incidence rates ranging from 0.01% [27] to 0.8% [26].

Numerous studies have examined the prevalence of Fabry disease in haemodialysis (HD) populations world-wide, with estimated prevalence ranging from 0.12% [28, 29] to 0.36% [30]. Fabry screening studies of Japanese HD populations [17, 31–35] estimate prevalence at 0.3 to 0.7% [32, 34, 35]. Prevalence studies of European HD populations estimate Fabry prevalence at between 0.15 and 0.36% [30, 36–38]. A Brazilian study estimated the prevalence of Fabry disease in haemodialysis patients to be 0.12% [28]. A recent Turkish study identified 17 cases of FD in male patients on renal replacement therapy (RRT), with 15 of those cases in patients who have had renal transplants (0.39%, *n* = 3882), two cases identified in patients on dialysis (0.12%, *n* = 1655), and an overall prevalence rate of 0.31% [29]. A systematic review of Fabry screening studies in high-risk populations revealed 12 studies of dialysis populations [39]. All 12 studies included men and 24 cases of FD were identified from a combined sample of 7182 men; an overall prevalence rate of 0.33%. Six of the studies examined prevalence of FD in women. Four FD cases were identified from a combined sample of 4719 females, yielding an overall prevalence estimate of 0.1%. Doheney et al. [40] conducted a reanalysis of 63 studies that estimated prevalence of Fabry disease in haemodialysis, renal transplant, stroke & cardiac populations with *GLA* mutations reported (total *n* = 51,363; 33,943 male, 17,420 female). Their revised prevalence estimate, after removal of those harbouring non-pathogenic *GLA* variants, for haemodialysis patients was 0.21% in males and 0.15% in females (*n* = 36,820; 23,954 males and 12,866 females). Revised prevalence rates for renal transplant patients was 0.24% males and 0.0% in females (*n* = − 3074; 2031 males and 1043 females).

Lack of consistent exclusion criteria across studies makes it difficult to calculate prevalence estimates. For example, some studies excluded previously identified FD cases [18]. Other studies excluded patients with a proven cause of renal failure [41, 42], which does not necessarily exclude FD. Each of the studies used alpha-Gal A analysis for initial screening, however the method varied with a combination of plasma, DBS, leucocytes and whole blood being used across the studies. This would lead to an underestimation of prevalence due to some cases of FD in female patients remaining unidentified. Different cut-off values were used in different studies, with higher thresholds prone to more false positive results. Most studies used DNA analysis to confirm diagnosis of FD, however at least one study did not [43].

The prevalence of FD in earlier chronic kidney disease (CKD) populations is less well studied, although rates of FD may be similar or related to the prevalence amongst ESKD patients. Two Turkish studies have examined the prevalence of FD in pre-terminal CKD patients, i.e. CKD patients not on RRT, Stages 1 to 5 [44, 45]. Alpha-Gal A testing using DBS was employed for both studies, with confirmation of diagnosis by genetic analysis. In both studies, FD was identified in male patients (prevalence of 1.8% [44] and 0.4% [45]) but in no female patients. Favalli et al. [46] examined the prevalence of Fabry disease in patients selected from multiple settings and identified that 2/72 CKD patients (2.7% prevalence) harboured a disease-associated GLA mutation, neither of whom were female. Despite no female patients with FD being identified in these three studies, this may be a result of the scale of screening (n~ 700 CKD patients), screening methods employed, or true lack of undiagnosed female FD cases. The aCQuire study will target a significantly larger screening sample (*n* = 3000) in company with a novel tiered screening approach to further explain this. Whilst the prevalence of FD is being explored in Australian dialysis populations [47] but the aCQuiRE Study is the first Australian study to look at FD prevalence in a broader CKD population.

### Screening and diagnosis

Symptom onset tends to be earlier for males, with symptoms beginning at around six to 10 years of age compared to nine to 13 years for females [15, 48–50]. Fabry disease symptomatology is non-specific and overlaps with that of other diseases. Misdiagnoses and diagnostic delays are common [49], with patients typically referred to several specialists before receiving a definite diagnosis [51]. Fabry Registry data indicates males are not diagnosed, on average, until about 24 years of age [15] and females remain undiagnosed until about 31 years of age [15]. These diagnostic delays can result in progressive, irreversible tissue damage [51]. In fact, 33% of males and 37% of females with Fabry disease are not diagnosed until after they reach end-stage renal disease (ESRD) [2]. Without treatment, males experience overt proteinuria and kidney failure during their 2nd to 5th decade [15]. Fabry Registry data indicated by 55 years of age, all surviving male Fabry patients with no detectable residual α-Gal A developed CKD [14].

Current Fabry screening options include Dried Blood Spot (DBS), Lyso-GB3 and genetic testing. DBS testing analyses alpha-galactosidase A (α-gal A) blood levels. It is easy and inexpensive to administer, and has been shown to be highly sensitive and specific for males. However, the α-gal A scores for females with FD overlap with the bottom of the normal range for women, and therefore may result in false negatives [52] which may occur in up to a third of affected females with FD [53]. Problems with sample quality can also lead to false positives [47, 54]. The Lyso-Gb3 test examines blood levels of globotriaosylsphingosine (Lyso-GB3). An assay using 0.01 mL of plasma yields high specificity (100%) for males and > 80% for females with classical FD, but is less reliable for non-classical mutations for both males and females [55]. DNA-based molecular genetic testing, involving sequencing of the *GLA* gene exons, is the gold standard for confirmation of Fabry diagnosis [54]. However, it is more costly and time-consuming than DBS and Lyso testing. Hence, the strategy employed is a broad screening strategy in men and women with CKD using tiered DBS, Lyso-GB3 and DNA testing, accepting that each of these modalities have diagnostic, operational and resource related factors. This pragmatic design accepts that the absence of screening may represent a less acceptable pathway than undertaking a screening approach that is implementable at scale in real-world clinical settings even in spite of identifiable limitations.

The α-gal A, Lyso-GB3 and *GLA* analysis employed will be clinically and diagnostically accredited (National Association of Testing Authorities, Australia), has been previously described [52, 55] and are detailed in Methods below.

### Treatment

Several treatments are available for FD. Three systematic literature reviews of Enzyme Replacement Therapy (ERT) have found significant decreases in GB3 accumulation and improvement in Fabry symptoms and quality of life for adult male [56], female [57] and paediatric Fabry populations [58]. ERT mimics the naturally-occurring α-Gal enzyme and breaks down GB3 throughout the body, including kidney cells, preventing further accumulation of GB3. Two preparations of ERT are currently prescribed for Fabry patients. Studies have found that these preparations can stabilise or improve Fabry symptomatology [59, 60]. Both preparations are funded under “Life Saving Drugs Program” in Australia.

Other therapies being developed include chaperone therapy, gene therapy, substrate reduction therapy, modified ERT and human gene therapy. Germain et al. [61] found that patients who took an oral pharmacological chaperone therapy showed no significant improvement in **glomerular filtration rate** (GFR**)** at 6 months compared to the placebo group. However, after controlling for male sex and high urinary protein excretion (which are predictive factors for faster progression to ESRD, changes at 24 months were similar to patients receiving ERT as published in Warnock, et al. [62]. Preclinical studies investigating systemic messenger RNA (mRNA) in mice and non-human primates support proof of concept for treatment of Fabry disease [63]. A multi-centre, phase I clinical trial of gene transfer therapy in men with FD (classical phenotype) has also been commenced [64]. Early indications from the first participant tentatively indicate that this therapy is feasible and safe but further results are awaited from additional participants.

### Aims

While the estimated prevalence of FD in the general and dialysis populations are known, little is known about its prevalence in the wider Chronic Kidney Disease (CKD) population. This study aims to determine the prevalence of Fabry disease at all stages of CKD. We hypothesise that screening of the Queensland CKD population with a blood-based enzyme/substrate strategy will identify unappreciated diagnoses of FD and assist in defining population prevalence. Additionally, individuals with CKD and FD may have a common set of features, which will assist in better targeting for future diagnostic resources and efforts. The prevalence of FD is being explored in Australian dialysis populations, but the aCQuiRE Study is the first Australian study to look at FD prevalence in a broader CKD population.

## Methods

### Study aim

The aim of the aCQuiRE Study is to determine the prevalence of Fabry disease in individuals with CKD stages 1–5/5D/5 T in Queensland, Australia and who receive care within a public health renal service. Further, the aCQuiRE Study has three broad objectives:

1. To identify individuals with CKD who have FD through the use of a blood-based enzyme/substrate testing and confirm through gene sequencing.
2. To characterise prevalence of affected individuals and establish if there are common disease presentations or characteristics which may assist in early diagnosis in other CKD populations.
3. To support identified individuals with genetic counselling and available treatment strategies, including ERT, and to identify and assist any affected family members.

### Study design

This is a prospective cohort screening study using a multi-tiered testing strategy amongst prevalent patients in Queensland affected by CKD and in public kidney health service care.

### Sites / target population

**Fig. 1:**
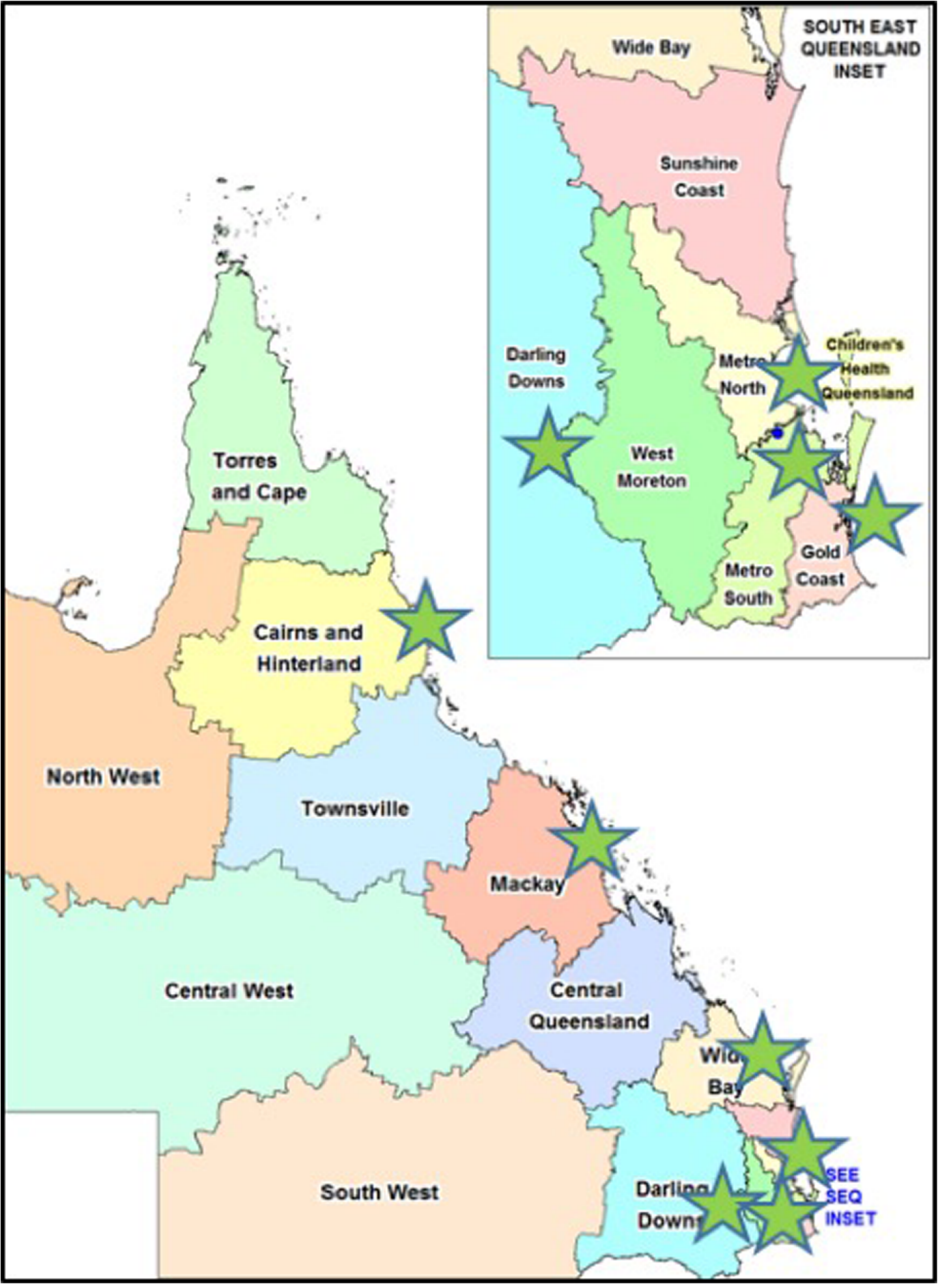
aCQuiRE Study Recruitment Sites in Queensland, Australia (Map Source: Queensland Health, https://www.health.qld.gov.au/maps, accessed 13 Dec 2019)

This study aims to screen 3000 patients with stages 1-5D/5 T CKD who are identified from the public renal speciality practices in Queensland, Australia. Patients for the screening study are being recruited from seven sites across Queensland, Australia: Kidney Health Service, Royal Brisbane and Women’s Hospital; Cairns Hospital; Logan Hospital; Toowoomba Hospital; Mackay Base Hospital; Hervey Bay Hospital; and Gold Coast University Hospital (Fig. 1). Study recruitment commenced in October 2017 and patients will be recruited until the end of August 2019 under a competitive recruitment model, where each site is able to recruit as many patients as possible until the number of 3000 patients is reached. Primary case follow-up is continuing until the end of October 2019. It is estimated that 0–1.5% (working assumption of 0.2%) of CKD patients, including those undergoing renal replacement therapy (dialysis or transplantation), will be affected by Fabry disease, and each of these patients is expected to have ~ 5 affected relatives. Out of the 3000 participants in this study, an estimated six index cases may be identified with potentially 30 affected relatives. Each site receives a reimbursement for nursing time involved in screening patients.

#### Patient/participant inclusion

To be eligible to participate, patients must be aged 18 years and over, a patient under the care of a renal specialist within Queensland Health (the public health system in Queensland), able to provide informed consent, and have chronic kidney disease stage 1-5D/5T (this includes patients who are undergoing dialysis and those who have had a renal transplant). Patients need to be eligible for Medicare so that they can access treatment if Fabry disease is indicated. Patients with a pre-existing Fabry disease diagnosis are eligible for inclusion though their participant is incidental and not targeted. Inclusion of such patients with a pre-existing Fabry diagnosis is to assist in defining a true prevalence of the condition amongst those with CKD, incorporating both those with a known or previously unknown diagnosis.Equal and balanced enrolment across sex and CKD stage will be monitored monthly and communicated to recruiting sites in monthly study progress update newsletters and second monthly site investigator meetings.

All eligible patients will be consecutively approached by their treating clinicians and/or site study coordinator as part of their standard clinically indicated contact with their treating kidney health service. A record of the number of approached patients declined participant and the number of total eligible patients at each recruitment site will be recorded. No additional details will be ascertained on such eligible patients declining or not undertaking consent.

#### Participant exclusion criteria

Patients are ineligible to participate if they are under 18 years of age; are not under the care of a renal specialist within Queensland Health; have cognitive impartment, intellectual disability or mental illness, and are unable to provide informed consent; or are not eligible for Medicare.

#### Patient/participant withdrawal

All patients provide informed, written consent prior to participation in the study. Patients may withdraw from the study at any time upon request, without any adverse consequences. When a participant withdraws, their individual data will be removed from the study (unless the patient specifies otherwise) and testing will be halted (if still in process) at the time withdrawal notification is received.

#### Clinical study registration

The aCQuiRE Study has been prospectively registered with the Queensland Health Database of Research Activity (DORA, https://dora.health.qld.gov.au) as pj09946 (Registered 3rd July 2017; https://dora.health.qld.gov.au/qldresearchjspui/cris/project/pj09946).

#### Availability of data and materials

The details, location and accessibility of the data which will be collected during this clinical study will be reported with the future peer-reviewed reporting of results. Those future results reporting and manuscript/s will reference this protocol article.

### Testing strategy

**Fig. 2:**
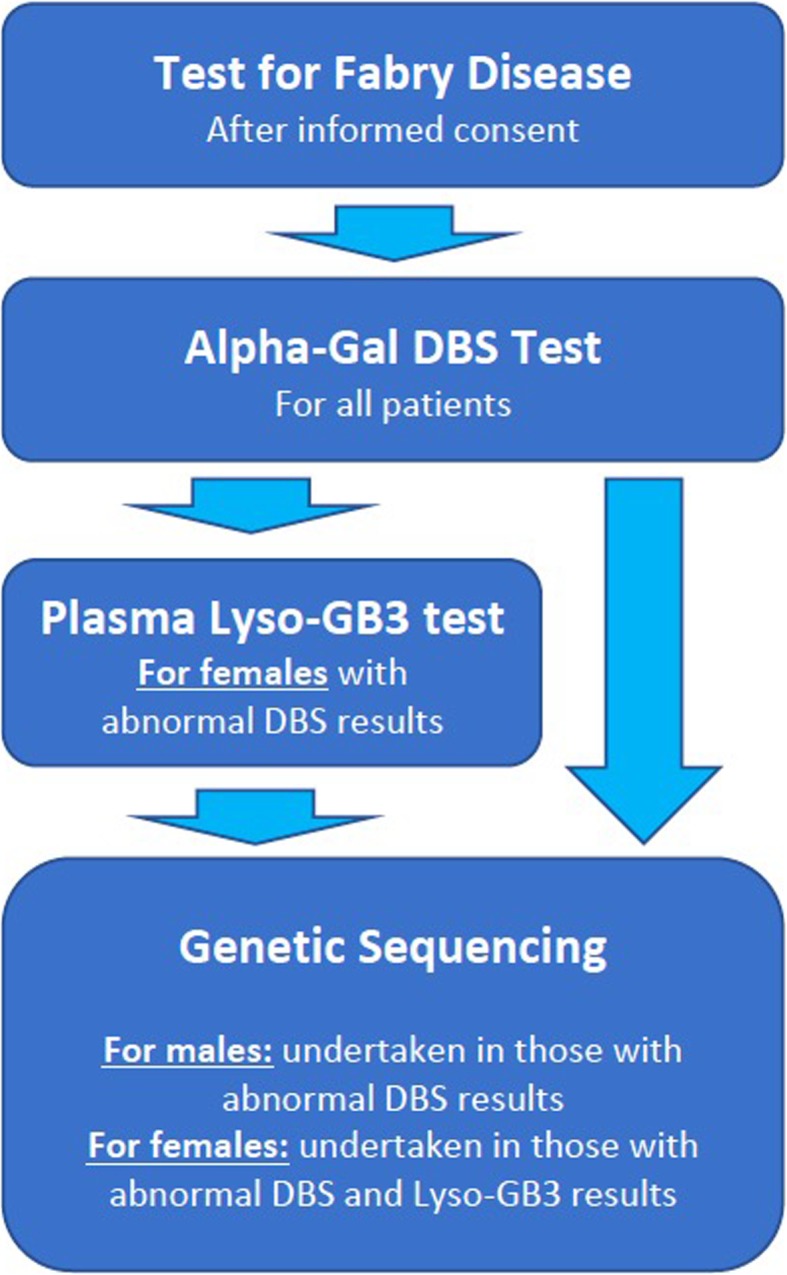
Screening Test Strategy for Fabry Disease in aCQuiRE Study

**Fig. 3:**
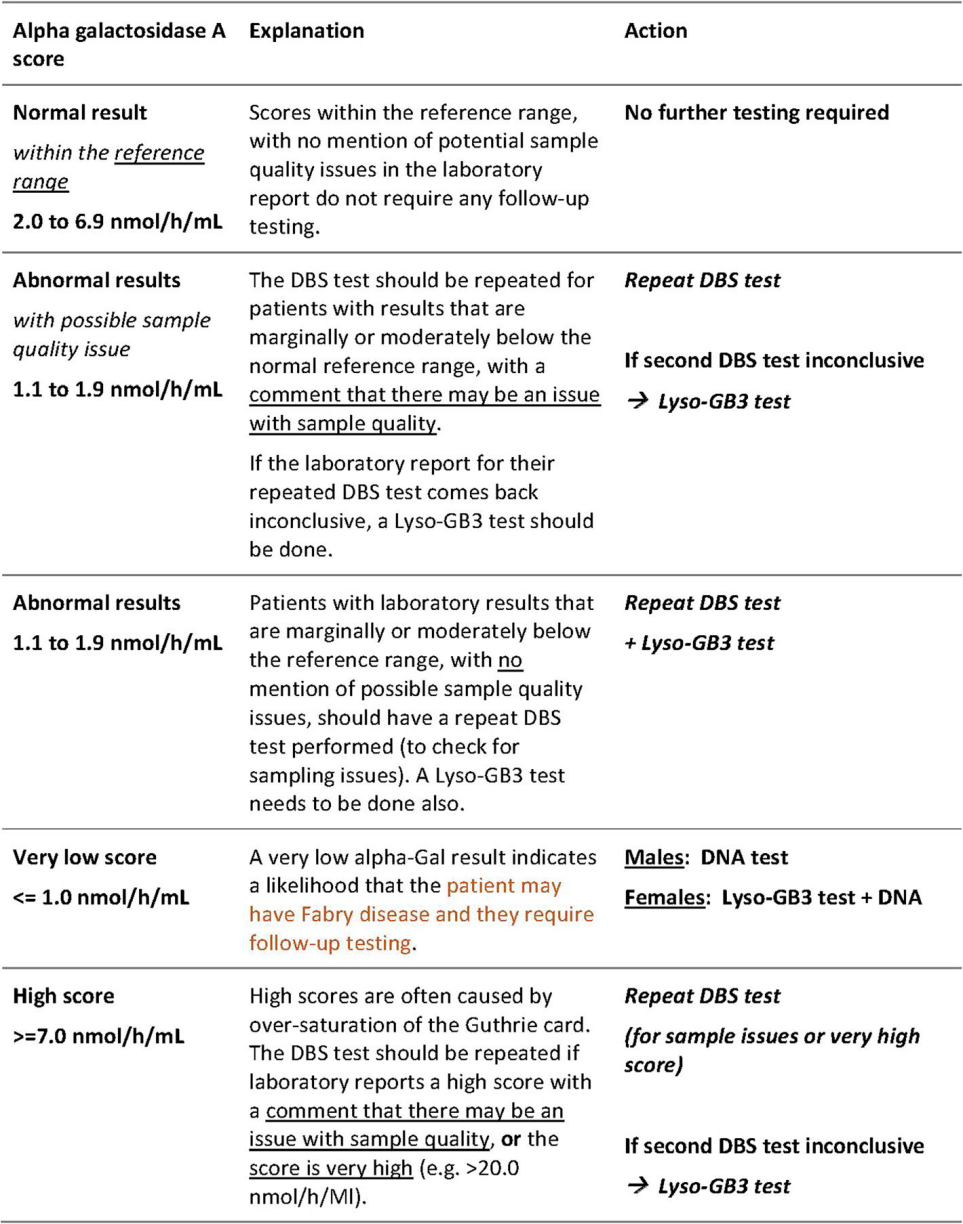
Interpretation of DBS α-Gal A Results in aCQuiRE Study

The aCQuiRE Study employs a cascade screening strategy comprising a combination of dried blood spot (DBS) testing, Lyso-GB3 testing and DNA sequencing (Fig. 2). In the context of the aCQuiRE study, cascade screening refers to sequential laboratory testing within the same participant rather than onward screening of at-risk family members. Females who receive abnormal DBS results (outside the reference range) undergo follow-up Lyso-GB3 testing concurrent with genetic testing. Males with abnormal DBS results have diagnosis confirmed through DNA testing. For those in whom a result returns with a reported sample quality issue, then the DBS is repeated anew. This includes in circumstances of a very high DBS result, which may reflect sample oversaturation. In both males and females in whom a marginal to moderately low DBS result returns without reporting of a sample quality issue, then the DBS is repeated in company with Lyso-GB3 testing, As per the gold standard, DNA-based (molecular genetic) testing/sequencing of the *GLA* gene exons is employed to confirm diagnoses of newly identified cases of FD. Where a diagnosis of FD is pre-existing, after the DBS, diagnosis is confirmed through Lyso-GB3 testing. Figure 3 shows the reference guide provided to sites for result reference ranges and follow-up testing under the cascade strategy.

#### Dried blood spot kits

Sites are provided with kits for collecting Dried Blood Spot (DBS) samples. The kits are comprised of a Guthrie card with a laboratory request form attached, lancet, alcohol swab, gauze, plaster, specimen bag, Reply Paid envelope, and humidity controller sachet.

#### Dried blood spot testing for α-Gal a

Dried Blood Spot (DBS) samples are collected by local clinical staff who are oriented to the study and to DBS testing procedures. Samples are collected on Guthrie paper, prior to dialysis and prior to the patient being heparinised, using the finger prick method. Guthrie cards are labelled with identifying information, as per standard diagnostic tests. The test is easy and inexpensive to administer and takes approximately 5 min. Samples are dried overnight and sent with a humidity controller sachet to the Genetics and Molecular Pathology Laboratory (SA Pathology) for analysis. A control enzyme (beta-galactosidase) is measured to ensure integrity of the blood spots. DBS Sampling Guidelines are followed precisely so that test results are accurate.

#### Lyso-Gb3 testing

Blood is collected for analysis of globotriaosylsphingosine (Lyso-GB3) in plasma. Two 4 ml EDTA tubes of blood are collected at the on-site Pathology Queensland laboratory. The samples are spun, aliquoted and the plasma frozen. The samples are batched and sent to the Genetics and Molecular Pathology Laboratory (SA Pathology) for analysis.

#### Genetic testing of GLA

Two 4 ml EDTA tubes of blood are collected at the on-site Pathology Queensland laboratory. The samples are sent to the central laboratory where DNA is extracted and stored. The extracted DNA is sent in batches to the Genetics and Molecular Pathology Laboratory (SA Pathology) for sequencing of the GLA gene.

### Reporting of findings, diagnosis and referral

**Fig. 4:**
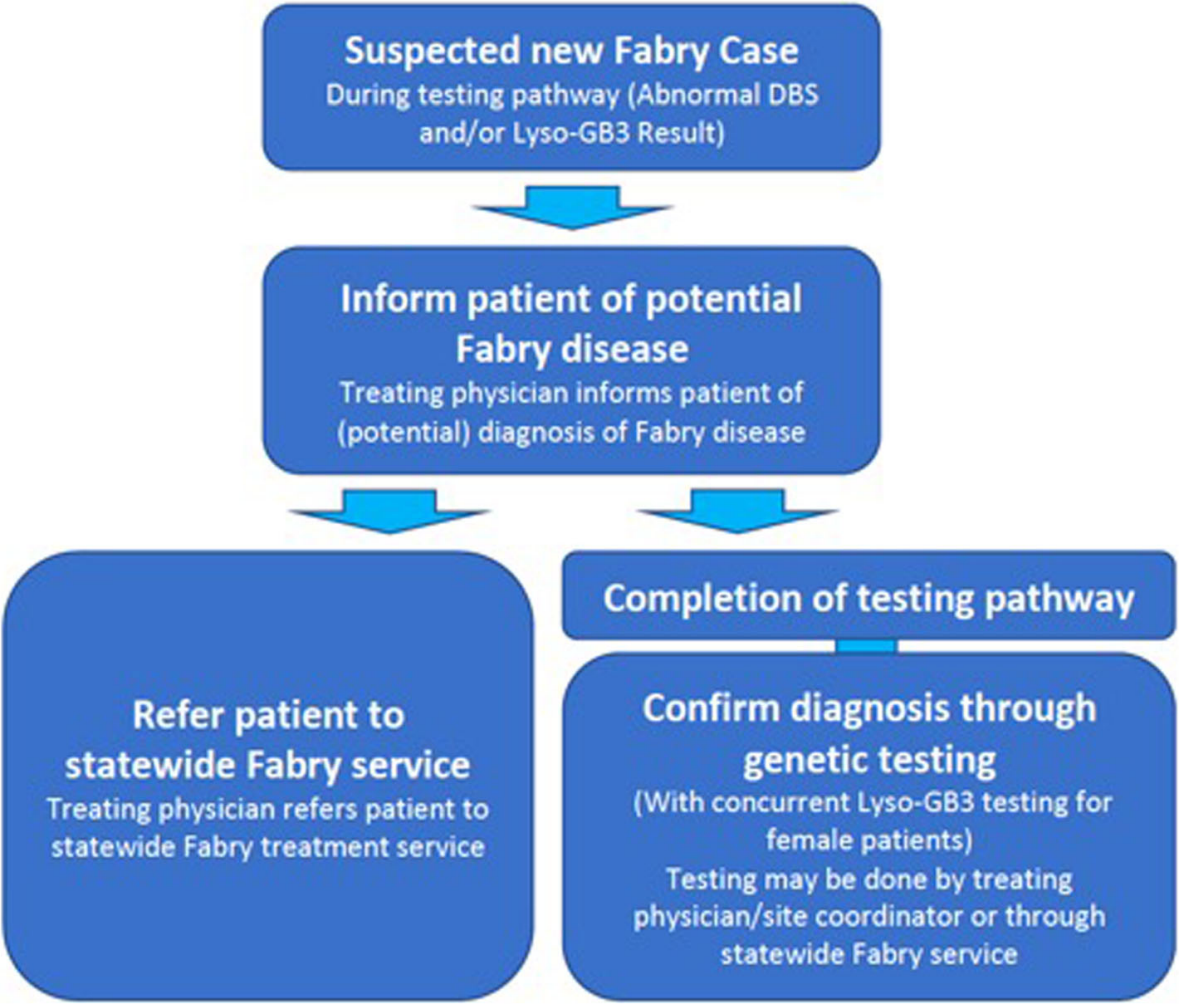
Reporting of Findings, Diagnosis and Referrals of Suspected Fabry Disease Cases in aCQuiRE Study

As shown in Fig. 4, where Fabry disease is indicated by laboratory results, the treating physician informs the patient of their diagnosis and offers referral to the Queensland Statewide Fabry Treatment Service (QSFTS). Confirmation of a diagnosis of Fabry disease is made through genetic sequencing. Enzyme replacement therapy is available under Medicare through this service. Genetic counselling is also provided and screening is available for family members.

#### Case report forms

Three case report forms were developed to capture patient demographics, relevant medical history and track screening, diagnosis and referral. The forms were based on current Fabry literature and consensus by study investigators (Supplementary Documents 1–3).

Case Report 1: *Patient Information and Clinical History* is used to collect patient demographics, such as sex, date of birth, Aboriginal and/or Torres Strait Islander status, South Sea Islander status, country of birth, as well as relevant medical history, relating to CKD stage, eGFR, proteinuria, and family history of kidney disease and Fabry disease.

Case Report 2: *Testing and Referral for Fabry Disease* tracks consent, testing and diagnosis of Fabry disease and referral to the QSFTS if indicated.

Case Report 3: *Health Issues and Symptoms* collects information about patients’ experience of symptoms found to be associated with Fabry disease under the following categories: Cardiac, Respiratory, Skin, Gastrointestinal Tract, Psychological, Renal, Cerebrovascular, Nervous System, Lymphoedema, Skeletal, Ocular and Other. An additional item asks the patient to rate their quality of life at the time of consent as “Good”, “Fair”, or “Poor”.

REDCap electronic data capture tools [65], hosted at The University of Queensland, are used to record data at each site under the supervision of the site coordinator, overseen by central administration. Data currently remains identified to allow for tracking of screening, diagnosis and referral.

All recruiting site coordinators and investigators will receive coordinated training in undertaking collection of patient information and symptoms. This training package will be undertaken by the same central program manager for all site coordinators and investigators and will occur at the site initiation visit and again at all monitoring visits.

### Statistical analysis

Mixed methods analysis of clinically and laboratory generated data will be employed for reporting of diagnoses of Fabry disease and description of clinical phenotype. Descriptive statistics will be used to compare the CKD cohort with Fabry disease to the CKD cohort without Fabry disease. The primary outcome will be the proportion of the screened population identified as having FD, with this subdivided into the proportion with a previously documented FD diagnosis or a new FD diagnosis. Additional variables that will be analysed include age, CKD stage and gender.

## Discussion

Expected outcomes of this study include more information about the prevalence of FD at all stages of CKD, including for females. This study may also provide information about common characteristics of Fabry disease that could assist with early diagnosis and optimal management/treatment. Diagnosis is important because Fabry disease is potentially treatable using enzyme replacement therapy, which may result in increased quality and duration of life.

Patients who are identified or suspected of having FD through the aCQuiRE study are offered referral to a statewide FD clinical service for assessment and direct care. ERT is available under Medicare to patients through this service. Being an X-linked disorder, other family members may be affected by Fabry disease and screening is also available through the service to relatives of diagnosed patients. Such screening provides opportunities for early diagnosis of Fabry disease and intervention/treatment before complications arise.

Strengths of this Fabry prevalence study include the capture of FD cases in both males and females and the inclusion of Fabry cases that have been previously identified. Challenges experienced have included issues with quality of DBS testing for a proportion of patients. Initially, some DBS samples were applied to the Guthrie card using a syringe. This method yielded a higher rate of sample quality issues and the need to collect repeat samples. The quality issue was thought to be related to blood spot volume and adherence to the process of using the finger prick method to collect peripheral blood has decreased the need for repeat sampling. However, there does remain the need for repeat sampling for some patients for a variety of reasons, including saturation of the Guthrie paper (as indicated by high results in the control), insufficient blood and marginally low alpha-Gal A levels for unknown reasons. Cascade testing, including a repeat DBS sample and/or Lyso-GB3 test, is utilised as an efficient way to resolve sampling issues.

While genetic testing has higher sensitivity for diagnosis of FD, it is much more costly and time-consuming compared to screening using DBS. We made the decision to use DBS sampling for initial testing to enable access to Fabry screening for a larger number of CKD patients, with follow-up Lyso-GB3 and genetic testing for confirmation of diagnosis as indicated. The inherent challenges imparted by the study methodologies may create challenges for achieving the primary aims of the study. These include applying a DBS screening test for all participants, both male and female, as well as targeting recruitment from a diverse geography of all stages of CKD. Nevertheless, risk management measures have been planned to closely monitor study progress to ensure robust recruitment, uniform data collection and methodological application of the screening test pathway. During the study, additional measures may be taken operationally should these potential risks become unveiled.

This is a significant and large study seeking to identify the prevalence of FD amongst patients with CKD of all stages across Queensland. While it examines prevalent rather than incident patient recruitment, an existing prevalent CKD cohort statewide initiative (CKD.QLD) [66] has resulted in significant and robust appreciation of prevalent cohort characteristics. This study will deploy a multi-cascade clinical testing strategy that intercalates both with local kidney health services as well as the statewide FD clinical service. Understanding of the prevalence of FD in this patient population will help to guide integration of FD screening into kidney health care provision, including identifying features suggesting those who might be at increased risk and timing of screening to ensure maximal patient benefit.

## Supplementary information

**Additional file 1: Supplementary Document 1.** *Case Report 1: Patient Information and Clinical History*

**Additional file 2: Supplementary Document 2.** *Case Report 2: Testing and Referral for Fabry Disease*

**Additional file 3: Supplementary Document 3.** *Case Report 3: Health Issues and Symptoms*

## Data Availability

The details, location and accessibility of the data which will be collected during this clinical study will be reported with the future peer-reviewed reporting of results. Those future results reporting and manuscript/s will reference this protocol article.

## Abbreviations

aCQuiRE Study: Ckd.Qld fabRy Epidemiology Study
CKD: Chronic Kidney Disease
DBS: Dry Blood Spot
DNA: Deoxyribonucleic Acid
**eGFR**: **Estimated Glomerular Filtration Rate** (CKD-EPI)
ERT: Enzyme Replacement Therapy
ESKD: End Stage Kidney Disease
FD: Fabry Disease
GB3: Globotriaosylceramide
GFR: **Glomerular Filtration Rate**
QSFTS: Queensland Statewide Fabry Treatment Service
α-Gal A: Alpha-galactosidase A
