# Supplementary material for: The Ckd. Qld fabRy Epidemiology (aCQuiRE) study protocol: identifying the prevalence of Fabry disease amongst patients with kidney disease in Queensland, Australia
Source: BMC Nephrol. 2020 Feb 22;21:58. doi: 10.1186/s12882-020-01717-9 (PMC7035781; doi:10.1186/s12882-020-01717-9)
Supplement: Supplementary file 1 — Additional file 1: Supplementary Document 1. Case Report 1: Patient Information and Clinical History [file 12882_2020_1717_MOESM1_ESM.docx]

**aCQuiRE Study**

**Case Report 1: Patient Information & Clinical History**

**Study Site**

🞎 RBWH, Metro North HHS 🞎 Darling Downs HHS

🞎 Logan Hospital, Metro South HHS 🞎 Mackay Hospital, Mackay HHS

🞎 Cairns & Hinterland HHS 🞎 GCUH, Gold Coast HHS

🞎 Harvey Bay Hospital, WBHHS

Please complete the survey below for the patient.

**Demographics & Consenting**

| Patient’s code UR# |  |
| --- | --- |
| Patient's last name |  |
| Patient's first name(s) |  |
| Patient's gender | 🞎 Male 🞎 Female |
| Patient's date of birth | / / (DD – MM - YYYY) |
| Date consent form signed | / / (DD – MM - YYYY) |
| Consulting nephrologist |  |
| Hospital /clinic name |  |
| Hospital UR |  |

**Please complete the patient’s clinical history on the back page of this form.**

**Clinical History**

| Does the patient have a family history of...  ...kidney Disease  ...Fabry disease | 🞎 Yes 🞎 No  🞎 Yes 🞎 No | |
| --- | --- | --- |
| Has the patient had a renal biopsy? | 🞎 Yes 🞎 No | |
| If yes, what were the results of the most recent renal biopsy? | 🞎 Copy of the results attached | |
| Does the patient have chronic kidney disease (CKD)? | 🞎 Yes 🞎 No | |
| If yes, what stage CKD does the patient have? | 🞎 Stage 1 | 🞎 Stage 4 |
|  | 🞎 Stage 2 | 🞎 Stage 5 |
|  | 🞎 Stage 3A | 🞎 Stage 5D |
|  | 🞎 Stage 3B | 🞎 Stage 5T |
| What is the most recent eGFR? | Date | |
| Does the patient have proteinuria? | 🞎 Yes 🞎 No | |
| If the patient has proteinuria, what were the last test results? | Date | |
|  |  | |
| Has the patient had RRT? |  | |
| If the patient has ESKD, how long has the patient been on RRT? | Date commenced | |
| What is the patient's modality of treatment? | 🞎 Haemodialysis  🞎 Peritoneal dialysis  🞎 Transplant | |
| If patient has had a renal transplant, was the donor: | 🞎 Living 🞎 Deceased | |
| If the patient has had a living transplant, was the donor related to the patient?  (Crosscheck with ANZ data) | 🞎 Not related  🞎 Related by marriage  🞎 Related by blood / genetically | |

Page 2
