# Supplementary material for: The Ckd. Qld fabRy Epidemiology (aCQuiRE) study protocol: identifying the prevalence of Fabry disease amongst patients with kidney disease in Queensland, Australia
Source: BMC Nephrol. 2020 Feb 22;21:58. doi: 10.1186/s12882-020-01717-9 (PMC7035781; doi:10.1186/s12882-020-01717-9)
Supplement: Supplementary file 2 — Additional file 2: Supplementary Document 2. Case Report 2: Testing and Referral for Fabry Disease [file 12882_2020_1717_MOESM2_ESM.docx]

**aCQuiRE Study**

**Case Report 2: Testing For Fabry Disease**

Please complete the survey below for the patient.

| Patient’s UR code |  |
| --- | --- |
| Patient's last name |  |
| Patient's first name(s) |  |
| Date dried blood sample (DBS) sent to SA Pathology | / / (DD – MM - YYYY) |
| Date DBS results received | / / (DD – MM - YYYY) |
| **α-Galactosidase activity level** |  |
| Lyso-GB3 test required | 🞎 Yes 🞎 No  (Tick 'Yes' if DBS test was inconclusive) |
| Date Lyso-GB3 sample sent to SA Pathology | / / (DD – MM - YYYY) |
| **Lyso-GB3 results** |  |
| DNA test required | 🞎 Yes 🞎 No  (Tick 'Yes' if the DBS/Lyso test was inconclusive) |
| Date DNA sample sent | / / (DD – MM - YYYY) |
| Date DNA test results received | / / (DD – MM - YYYY) |
| **DNA test results** |  |
| Are the DNA test results positive for Fabry disease? | 🞎**Fabry disease** 🞎Not FD 🞎Inconclusive |
| If referred, date referred to genetic counselling. | / / (DD – MM - YYYY)  (If the patient tests positive to the Fabry DNA test, he/she should be referred to the Qld Statewide Fabry Treatment Service (QSFTS) for treatment and/or genetic counselling.) |
| What is the number of patient’s surviving, first degree relatives that may be at risk of having Fabry disease? | 🞎 # parents  🞎 # siblings  🞎 # children  🞎 # nieces and nephews  **🞎 Total # at-risk relatives** |
| Comments |  |
