# Supplementary material for: The Ckd. Qld fabRy Epidemiology (aCQuiRE) study protocol: identifying the prevalence of Fabry disease amongst patients with kidney disease in Queensland, Australia
Source: BMC Nephrol. 2020 Feb 22;21:58. doi: 10.1186/s12882-020-01717-9 (PMC7035781; doi:10.1186/s12882-020-01717-9)
Supplement: Supplementary file 3 — Additional file 3: Supplementary Document 3. Case Report 3: Health Issues and Symptoms [file 12882_2020_1717_MOESM3_ESM.docx]

**aCQuiRE Study**

AFFIX PATIENT LABEL HERE

**Case Report 3: Health Issues & Symptoms**

**1. Study Site**

🞎 Kidney Health Service, Metro North HHS 🞎 Darling Downs HHS

🞎 Logan Hospital, Metro South HHS 🞎 PA Hospital, Metro South HHS

🞎 Cairns & Hinterland HHS 🞎 Other

**2. Patient Details**

| Patient’s UR# |  |
| --- | --- |
| Patient's last name |  |
| Patient's first name(s) |  |

**3. Health Issues / Symptoms**

Please ask the patient if they have **ever experienced** the following symptoms/health issues and tick the box under the under the column headed “Patient-reported” for all that apply.

Next, check the patient’s medical file and tick the box under the column headed “Verified from patient file" for all symptoms/health issues noted in the patient’s file.

Add any extra relevant information under the “Comments” column.

| **A) Cardiac** | **Patient- reported** | **Verified from patient file** | **Comments** |
| --- | --- | --- | --- |
| 1. Heart attack | 🞏 | 🞏 |  |
| 1. Heart failure | 🞏 | 🞏 |  |
| 1. Chest pain | 🞏 | 🞏 |  |
| 1. Palpitations | 🞏 | 🞏 |  |
| 1. Fainting, passing out | 🞏 | 🞏 |  |

| **B) Respiratory** | **Patient- reported** | **Verified from patient file** | **Comments** |
| --- | --- | --- | --- |
| 1. Shortness of breath on exertion | 🞏 | 🞏 |  |
| 1. Shortness of breath lying flat | 🞏 | 🞏 |  |
| 1. Cough | 🞏 | 🞏 |  |
| 1. Wheeze | 🞏 | 🞏 |  |

| **C) Skin** | **Patient- reported** | **Verified from patient file** | **Comments** |
| --- | --- | --- | --- |
| 1. Skin rash (such as angiokeratoma) | 🞏 | 🞏 |  |

| **D) Gastrointestinal Tract** | **Patient- reported** | **Verified from patient file** | **Comments** |
| --- | --- | --- | --- |
| 1. Abdominal pain, bloating and diarrhoea | 🞏 | 🞏 |  |
| 1. Nausea, vomiting | 🞏 | 🞏 |  |
| 1. Difficulty gaining weight | 🞏 | 🞏 |  |

| **E) Psychological** | **Patient- reported** | **Verified from patient file** | **Comments** |
| --- | --- | --- | --- |
| 1. Depression | 🞏 | 🞏 |  |
| 1. Anxiety | 🞏 | 🞏 |  |
| 1. Other | 🞏 | 🞏 |  |

iv) How does the patient rate their quality of life?

🞎 Good 🞎 Fair 🞎 Poor

| **F) Renal** | **Patient- reported** | **Verified from patient file** | **Comments** |
| --- | --- | --- | --- |
| Albuminuria, proteinuria |  |  | 🞎 None/minimal  🞎 Mild  🞎 Severe |
| Kidney disease | 🞏 | 🞏 |  |
| Kidney failure | 🞏 | 🞏 |  |
| Scant / absent urine | 🞏 | 🞏 |  |
| Unknown | 🞏 | 🞏 |  |

| **G) Cerebrovascular:** | **Patient- reported** | **Verified from patient file** | **Comments** |
| --- | --- | --- | --- |
| Stroke/ Transient Ischaemic Attacks (TIAs) | 🞏 | 🞏 |  |

| **H) Nervous System:** | **Patient- reported** | **Verified from patient file** | **Comments** |
| --- | --- | --- | --- |
| Peripheral neuropathic pain and/or acroparathesias | 🞏 | 🞏 |  |
| Vertigo | 🞏 | 🞏 |  |
| Tinnitus | 🞏 | 🞏 |  |
| Hearing loss and/or nerve deafness | 🞏 | 🞏 |  |
| Hypohidrosis (decreased sweating) | 🞏 | 🞏 |  |
| Heat intolerance, heat stroke and/or reduced exercise capacity | 🞏 | 🞏 |  |

| **Lymphoedema:** | **Patient- reported** | **Verified from patient file** | **Comments** |
| --- | --- | --- | --- |
| Lymphoedema | 🞏 | 🞏 |  |

| **Skeletal:** | **Patient- reported** | **Verified from patient file** | **Comments** |
| --- | --- | --- | --- |
| Osteopenia | 🞏 | 🞏 |  |
| Osteoporosis | 🞏 | 🞏 |  |

| **Ocular:** | **Patient- reported** | **Verified from patient file** | **Comments** |
| --- | --- | --- | --- |
| Corneal and/or lens opacities (cataracts) | 🞏 | 🞏 |  |
| Vasculopathy of retina and/or conjunctiva | 🞏 | 🞏 |  |

| **Other:** | **Patient- reported** | **Verified from patient file** | **Comments** |
| --- | --- | --- | --- |
|  | 🞏 | 🞏 |  |
